# Prognostic Impact of Psoas Muscle Mass Index following Trans-Catheter Aortic Valve Replacement

**DOI:** 10.3390/jcm12123943

**Published:** 2023-06-09

**Authors:** Teruhiko Imamura, Hayato Fujioka, Ryuichi Ushijima, Mitsuo Sobajima, Nobuyuki Fukuda, Hiroshi Ueno, Koichiro Kinugawa

**Affiliations:** The Second Department of Internal Medicine, University of Toyama, Toyama 930-0194, Japan; tbman2@gmail.com (H.F.); ryuushi@med.u-toyama.ac.jp (R.U.); sobajima@med.u-toyama.ac.jp (M.S.); nfukuda@med.u-toyama.ac.jp (N.F.); hueno@med.u-toyama.ac.jp (H.U.); kinugawa-tky@umin.ac.jp (K.K.)

**Keywords:** heart failure, hemodynamics, sarcopenia, aortic valve disease

## Abstract

Background: Psoas muscle mass is a recently featured index of sarcopenia, which has a negative prognostic impact in patients with a variety of diseases. We investigated the prognostic impact of baseline psoas muscle mass in patients receiving a trans-catheter aortic valve replacement (TAVR). Methods: Patients who received TAVR at our center between 2015 and 2022 were included. Patients received computer tomography imaging upon admission as an institutional protocol, and psoas muscle mass was measured, which was indexed by body surface area. Patients were followed for four years or until January 2023. The prognostic impact of psoas muscle mass index on 4-year mortality following index discharge was evaluated. Results: A total of 322 patients (85 years, 95 male) were included. Median psoas muscle mass index at baseline was 10.9 (9.0, 13.5) × 10 cm^3^/m^2^. A lower psoas muscle mass index tended to be associated with several index of malnutrition and sarcopenia. A psoas muscle mass index was independently associated with 4-year mortality with an adjusted hazard ratio of 0.88 (95% confidence interval 0.79–0.99, *p* = 0.044). Patients with lower psoas muscle mass index (below the statistically calculated cutoff of 10.7 × 10 cm^3^/m^2^, N = 152) had significantly higher cumulative 4-year mortality compared with others (32% versus 13%, *p* = 0.008). Conclusions: A lower psoas muscle mass index, which is a recently featured objective marker of sarcopenia, was associated with mid-term mortality following TAVR in the elderly cohort with severe aortic stenosis. The measurement of psoas muscle mass index prior to TAVR could have clinical implications for shared decision-making among patients, their relatives, and clinicians.

## 1. Background

Trans-catheter aortic valve replacement (TAVR) was introduced as a less invasive trans-catheter intervention to treat severe aortic stenosis for patients requiring surgical valve replacement. Clinical outcomes following TAVR, including the safety of the procedure and reduction in procedure-related complications, have improved significantly due to optimal patient selection, the establishment of dedicated imaging analyses for pre-procedural planning, the introduction of a new generation device design, minimization of the procedure including single arterial access and conscious sedation, and the transition from dual to single antiplatelet therapy for post-TAVR medication [1,2]. The indication for TAVR has expanded to include intermediate and lower risk patients, leading to an expansion of the current guideline recommendation for TAVR.

However, some patients still experience a higher rate of mortality after TAVR [3]. Many TAVR candidates are elderly patients with frailty and sarcopenia because aortic stenosis progresses in patients with worsening atherosclerosis and vale degeneration [4]. These comorbidities are associated with worse clinical outcomes in patients with a variety of conditions, including heart failure and valvular disease [5]. Hemodynamics can be ameliorated by TAVR, but these comorbidities are not necessarily improved after successful TAVR alone. Risk stratification by assessing baseline parameters associated with these comorbidities could be useful for shared decision-making prior to TAVR, especially in elderly patients with multiple comorbidities.

Their negative prognostic impact has been reported in patients undergoing TAVR [6,7,8]. However, there is debate as to which criteria best define frailty and sarcopenia and risk stratify patient cohorts, given the complexity of defining these diseases. Several available clinical scales have limitations because they are subjective, semi-quantitative, and complex to calculate, making them difficult for routine clinical use in most general centers.

Sarcopenia is defined as a decrease in muscle strength or muscle mass [9]. Thus, sarcopenia has the potential to be objective and quantitative. With improvements in imaging technology, muscle mass can be quantified by using computed tomography or magnetic resonance imaging [10]. A variety of methods have been proposed to assess muscle mass. Recently, psoas muscle mass volume (instead of skeletal muscle area at the level of the third lumbar vertebra or psoas muscle square) has been proposed as a novel index to assess the degree of sarcopenia and to risk stratify patients with malignancy [11,12]. Overall, in this study, we attempted to evaluate the prognostic impact of psoas muscle mass in elderly patients with severe aortic stenosis undergoing TAVR. 

## 2. Methods

### 2.1. Patient Selection

Patients with severe aortic stenosis who underwent TAVR at our center between 2015 and 2022 were included. Patients received computed tomography imaging upon admission according to our institutional protocol. Patients who lacked clinical data, including computed tomography imaging, were excluded. Patients who died during index hospitalization were excluded given the lack of follow-up data. Patients who lost their lives before follow-up were also excluded. 

Written informed consent was obtained from all participants upon admission. The institutional review board approved the study protocol. 

### 2.2. Measurement of Psoas Muscle Mass

**Figure 1:**
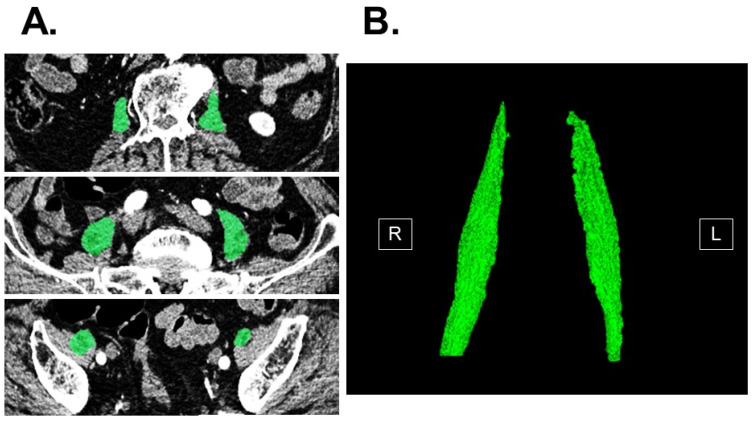
Measurement of total psoas muscle mass volume using abdominal computed tomography imaging. Bilateral psoas muscles (green) in horizontal section were automatically traced by the software in each slice (**A**). All slices were combined and three-dimensional image of bilateral psoas muscle with calculated volume was obtained (**B**).

Computed tomography imaging obtained upon admission according to the standard manner was used to measure psoas muscle mass volume using SYNAPSE VINCENT software version 2.0003 (Fujifilm, Tokyo, Japan). Three-dimensional abdominal imaging was re-constructed by using multi-sliced computed tomography data (green areas indicate psoas muscle mass in Figure 1) [13,14]. The software automatically traced the psoas muscle and calculated psoas muscle mass volume. Before final calculation, an experienced researcher confirmed and modified the trace as appropriate.

### 2.3. TAVR Procedure

Patients with severe aortic stenosis with peak velocity >4.0 m/s, mean pressure gradient >40 mmHg, or aortic valve area <1.0 cm^2^ were eligible for TAVR. The indication for TAVR was determined by clinical consensus of a multidisciplinary team including cardiac surgeons, interventional cardiologists, anesthesiologists, and imaging specialists. Patients underwent standard TAVR procedure using the Edwards Sapien XT/Sapien 3 Transcatheter Heart Valve (Edwards Lifesciences, Irvine, CA, USA) or the Medtronic CoreValve/Evolut R Revolving System (Medtronic, Minneapolis, MN, USA). TAVR were performed via trans-femoral, trans-subclavian, or direct-aorta under local/systemic anesthesia. 

An antithrombotic regimen following TAVR was used at the discretion of the physician. Patients were discharged after being carefully observed in hospital for one week and the confirmation of no active procedure-related complications. Following index discharge, patients were followed-up at our out-patient clinic or affiliated institutions at scheduled intervals by board-certified cardiologists. Standard laboratory and echocardiographic data were followed at least once per year.

### 2.4. Independent Variable and the Primary Outcome

An independent variable was defined as psoas muscle mass index, which was adjusted by body surface area (Du Bois method), measured prior to TAVR. The primary outcome was defined as a four-year mortality after index discharge. 

### 2.5. Other Clinical Variables

Demographic, comorbidity, laboratory, and echocardiographic data obtained at index discharge after TAVR were collected as baseline characteristics. Frailty data, including mini-mental state, Canadian study of health and aging scale, specific activity scale, and Barthel index, were calculated at index discharge. Day 0 was defined as the time of index discharge (patients who died during the index hospitalization were excluded due to the lack of follow-up data). 

### 2.6. Statistical Analysis

Continuous variables were presented as median and interquartile range and compared using Mann–Whitney U test considering a moderate sample size. Categorical variables were presented as numbers and percentages and compared using Fisher’s exact test. A value of two-tailed *p* < 0.05 was considered statistically significant. Statistical analyses were performed using SPSS Statistics 22 (SPSS Inc., Armonk, IL, USA). 

An independent variable was defined as baseline psoas muscle mass index and a primary outcome was defined as four-year mortality after TAVR. Day 0 was the time of index discharge. 

Cox proportional hazard ratio regression analyses were performed to investigate the prognostic impact of baseline characteristics, including psoas muscle mass index, which was obtained after TAVR unless otherwise stated. Variables that were significantly different between deceased and alive patients were included in the Cox analyses. Only variables significant in the univariable analyses were included in the multivariable analysis. 

Receiver operating characteristics analysis was performed to calculate a cutoff of psoas muscle mass index for the primary outcome. Patients were stratified by the cutoff. Kaplan–Meier curves were compared between the two groups by log-rank test. 

## 3. Results

### 3.1. Baseline Characteristics at Index Discharge

A total of 345 patients who underwent TAVR at our center between 2015 and 2022 were eligible for this study. Patients who died during the index hospitalization and those with missing data were excluded. In the end, 322 patients were included. Median age was 85 years, and 95 were male. Coronary artery disease was observed in 87 (27%) patients, and 42 (13%) patients had atrial fibrillation. Prior to TAVR, median peak velocity at aortic valve was 4.4 (4.0, 4.9) m/s, left ventricular end-diastolic diameter was 45 (41, 51) mm, and left ventricular ejection fraction was 64% (54%, 70%).

Following TAVR, median serum albumin was 3.4 (3.1, 3.6) g/dL, and median estimated glomerular filtration rate was 51 (37, 64) mL/min/1.73 m^2^. Median peak velocity at aortic valve was 2.2 (1.8, 2.4) m/s. Median mini-mental state was 26 (23, 28), and median Canadian Study of Health and Aging (CSHA) scale was 4 (3, 4). 

### 3.2. Prognostic Impact of Baseline Characteristics including Psoas Muscle Volume

During the 4-year observation period after the index discharge (median 760 days), 36 patients died. The causes of death were varied. The most common cause of death was senility (N = 20). Other causes of death were as follows: three due to pneumonia, three due to renal failure, three due to infection, two due to sudden death, two due to heart failure, one due to malignancy, one due to stroke, and one due to hepatic failure. Patients who died had a higher STS score at baseline (Table 1). Peripheral artery disease and atrial fibrillation were more prevalent in the deceased patients. Plasma B-type natriuretic peptide at baseline was higher in the deceased patients. The severity of frailty was more advanced with a higher CSHA scale in the deceased patients.

**Figure 2:**
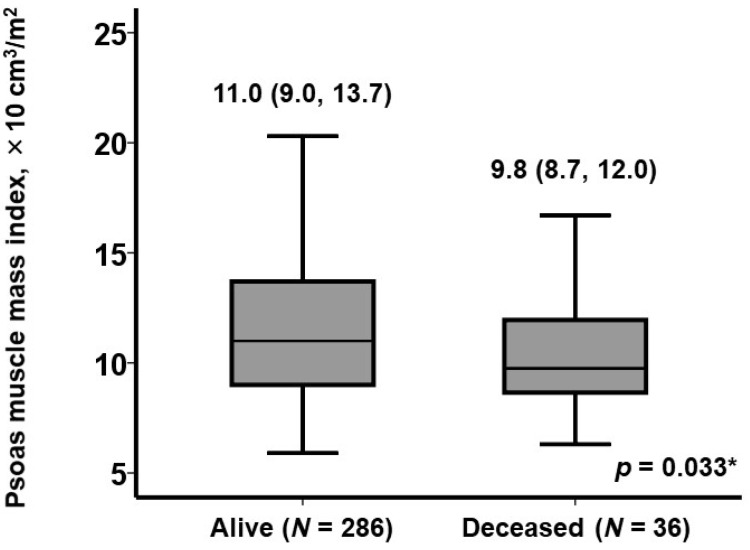
Psoas muscle mass index between deceased and living patients after TAVR. Psoas muscle mass index was reported as median and interquartile range and compared between the two groups using Mann–Whitney U test. * *p* < 0.05.

Median psoas muscle mass index was 10.9 (9.0, 13.5) × 10 cm^3^/m^2^. Deceased patients had lower psoas muscle mass index at baseline than living patients (9.8 [8.7, 12.0] versus 11.0 [9.0, 13.7] × 10 cm^3^/m^2^, *p* = 0.033; Figure 2). 

Five variables that were significantly different between deceased and living patients were included in the time-to-event analysis, including the psoas muscle mass index (Table 2). Baseline psoas muscle mass index (per 10 cm^3^/m^2^) was independently associated with 4-year mortality with an adjusted hazard ratio of 0.88 (95% confidence interval 0.79–0.99, *p* = 0.044).

**Figure 3:**
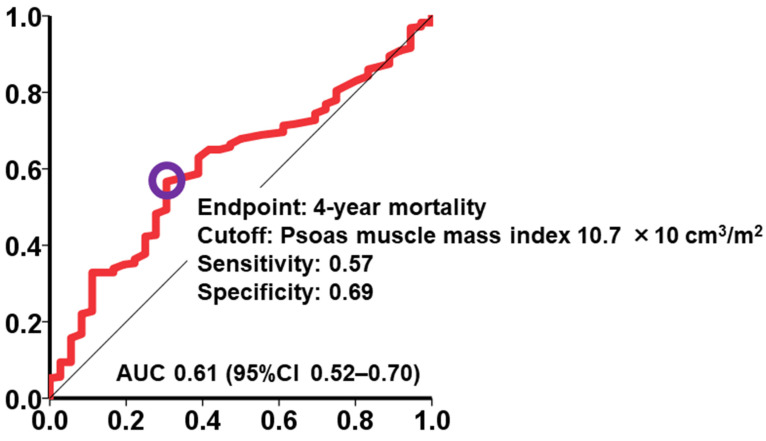
Psoas muscle mass index cut-off value for predicting 4-year mortality after TAVR. AUC, area under the curve.

**Figure 4:**
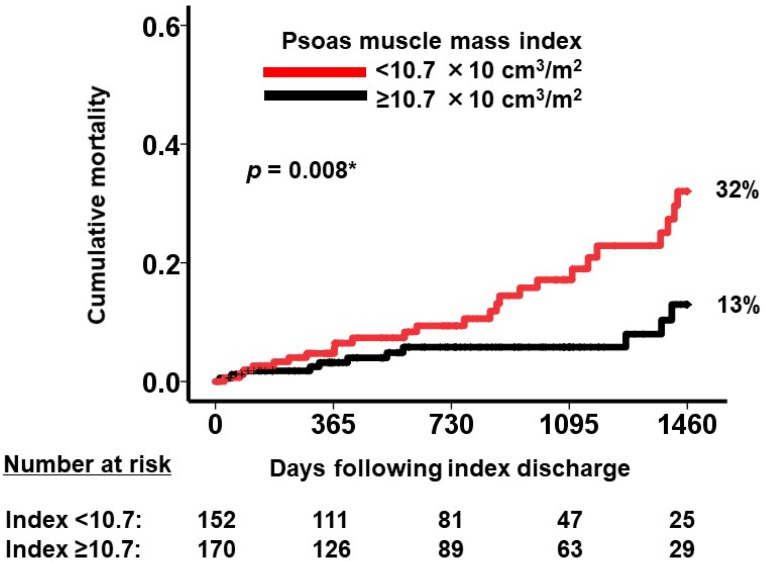
Four-year cumulative mortality stratified by psoas muscle mass index cutoff. * *p* < 0.05 by log-rank test. A lower psoas muscle mass index below the cutoff was associated with higher four-year cumulative mortality.

A cutoff value for psoas muscle mass index to best stratify the primary outcome was calculated as 10.7 × 10 cm^3^/m^2^ with a sensitivity of 0.57 and a specificity of 0.69 (Figure 3). Patients with psoas muscle mass index <10.7 × 10 cm^3^/m^2^ had significantly higher cumulative 4-year cumulative mortality compared to those with ≥10.7 × 10 cm^3^/m^2^ (32% versus 13%, *p* = 0.008; Figure 4). A hazard ratio of a dichotomized psoas muscle mass index <10.7 × 10 cm^3^/m^2^ was 2.52 (95% confidence interval 1.24–5.12, *p* = 0.011).

### 3.3. Profile of Patients with Low Psoas Muscle Mass

Patient characteristics were stratified by the cutoff of psoas muscle mass index to show the profile of patients with low psoas muscle mass (Table 3). Patients with smaller psoas muscle mass index had higher body mass index, lower hemoglobin, and lower total cholesterol compared to those with larger psoas muscle mass (*p* < 0.05 for all).

**Figure 5:**
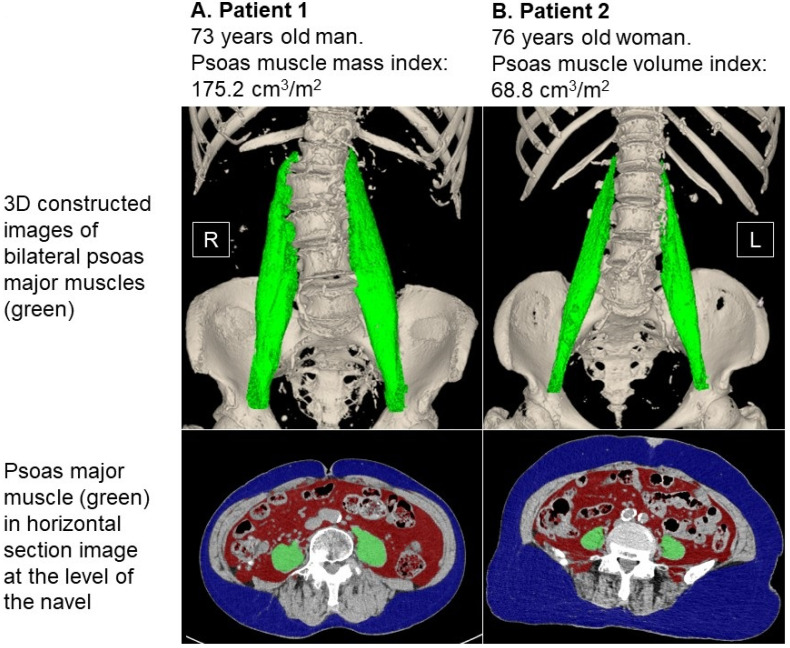
Representative cases of high psoas muscle mass index (**A**) and low psoas muscle mass index (**B**). Patient 1 was a 73-year-old man. His body height was 166 cm, and his body weight was 58.4 kg. His psoas muscle mass index was calculated to be 175.2 cm^3^/m^2^. Patient 2 was a 76-year-old woman. Her body height was 147 cm, and her body weight was 51.3 kg. Her psoas muscle mass index was calculated to be 68.8 cm^3^/m^2^. Psoas muscle mass (highlighted in green) appears larger in patient 1 compared to patient 2 in both three-dimensional and two-dimensional imaging.

Typical cases with small and large psoas muscle mass index are displayed in Figure 5A,B. A 73-year-old male patient had 166 cm of body height and 58.4 kg of body weight (body mass index was 21.2). His psoas muscle mass was 288.5 cm^3^, and it was indexed as 175.2 cm^3^/m^2^ (Figure 5A). Another 76-year-old female patient had 147 cm of body height and 51.3 kg of body weight (body mass index 23.8). Her psoas muscle mass was 98.2 cm^3^, and it was indexed as 68.8 cm^3^/m^2^ (Figure 5B).

## 4. Discussion

In this study, we investigated the prognostic impact of baseline psoas muscle mass, which was indexed by patients’ body surface area, in patients with severe aortic stenosis undergoing TAVR. Psoas muscle mass index was widely distributed. Psoas muscle mass index was an independent prognostic factor for 4-year mortality after TAVR at a cutoff of 10.6 × 10 cm^3^/m^2^. A lower psoas muscle mass was associated with several worse baseline characteristics, including anemia.

### 4.1. Psoas Muscle Mass and Sarcopenia

An increasing number of elderly patients with multiple comorbidities are now receiving interventional therapies, including TAVR [15]. Optimal patient selection, accurate prognostic stratification, and appropriate shared decision-making are of great importance in such an era. Otherwise, unwilling and excessive interventional therapies could decrease patient satisfaction, worsen the quality of life among patients, and shorten the prognosis of patients. One of the most prominent risk factors for such a cohort receiving TAVR is sarcopenia [8]. Hemodynamics can be improved via the anatomical correction of the stenotic aortic valve by TAVR, but sarcopenia can persist even after hemodynamic improvement by successful TAVR. Sarcopenia is strongly associated with frailty and malnutrition [16]. Of these, sarcopenia is relatively more objective than others and may be a promising tool for risk stratification.

Sarcopenia is commonly assessed by measuring skeletal muscle mass using computed tomography or magnetic resonance imaging, but detailed methodology, including which muscle to measure and how to adjust it, remains unestablished [9]. Traditionally, the skeletal muscle index, which is the indexed cross-sectional total muscle area at the horizontal height of L3, has been used to assess the degree of sarcopenia [17]. The index had a strong correlation with other traditional indices, including the dual-energy X-ray absorptiometry method. Psoas mass area has been demonstrated to have a strong correlation with skeletal muscle index [18]. Recently, given the improvement of technology, three-dimensional psoas muscle volume has been proposed as an alternative to the above conventional index. The psoas muscle volume is strongly correlated with the cross-sectional total muscle area at the horizontal height of L3, and its decrease with age, as with dual-energy X-ray absorptiometry and bioelectrical impedance analysis muscle mass measurements, has also been observed [19].

Consistently, a lower psoas muscle mass index tended to be associated with malnutrition and frailty parameters in this study. A lower psoas muscle mass volume index was associated with a higher body mass index (instead of a lower body mass index), as also displayed in Figure 5A,B, which may be explained by the concept of sarcopenic obesity [20]. The psoas muscle plays an important role in maintaining an appropriate gait. Atrophic psoas muscle impairs daily activity, leading to frailty among patients [21].

In a recent study conducted abroad, obesity was associated with lower in-hospital mortality, cardiogenic shock, and bleeding complications after TAVR [22]. Median BMI in our study was 21.7, and few patients had obesity. Most of the TAVR candidates in our study had cardiac cachexia with advanced sarcopenia. Obesity patients may have sufficient nutritious status and less advanced sarcopenia, resulting in favorable clinical outcomes after TAVR. Although no data have been shown, if measured in this study, we speculate that psoas muscle mass index may be high in the obese patients.

### 4.2. Prognostic Impact of Psoas Muscle Mass following TAVR

Many TAVR candidates are older and have multiple comorbidities. As a result, they tend to have advanced sarcopenia. Consistently, the median psoas muscle mass index was 10.9 × 10 cm^3^/m^2^, which is lower compared to results reported elsewhere in the literature [23].

Hemodynamics almost improve after valve repair with TAVR, and it is perhaps not surprising that baseline hemodynamics parameters were not significant predictors of mortality. Instead, sarcopenia, which is generally associated with worse clinical outcomes, would persist even after TAVR because sarcopenia is not a direct target of TAVR. Therefore, it is not surprising that psoas muscle mass was a significant predictor of mortality. Most of the causes of death were related to sarcopenia rather than heart failure. Recent studies showing anti-inflammatory and anti-apoptotic effects of skeletal muscle also help to explain our findings [24]. Patients with reduced skeletal muscle may have less chance to enjoy these favorable benefits.

Other previous studies have also showed the short-term prognostic impact of skeletal muscle size in the TAVR candidates, including dominant psoas muscle area and skeletal muscle mass index [23,25,26]. In light of recent studies being added to the literature, psoas muscle mass, which is measured according to three-dimensional muscle volume, would be superior to these conventional variables. Other frailty parameters were not significant predictors in this study, probably because they are subjective or semi-objective.

The measurement of psoas muscle mass is practical. Candidates for TAVR routinely undergo pre-procedural computed tomography imaging to assess the severity of aortic stenosis and their vascular anatomy to guide the procedural strategy. They do not need additional testing to obtain this novel index. We can easily calculate psoas muscle mass volume using commonly available software from routinely obtained computed tomography imaging prior to TAVR.

We do not conclude that candidates with psoas muscle mass index below the cutoff should not receive TAVR simply from our findings. Even patients with reduced skeletal muscle can enjoy greater clinical benefits from TAVR as opposed to just receiving medical therapy alone. Instead, we recommend using our findings for shared decision-making between clinicians, TAVR candidates, and their relatives. The applicability of our findings is to be prospectively validated in our next study. Pre-procedural cardiac exercise may increase psoas muscle mass and result in greater prognosis post-TAVR. However, such an aggressive exercise is contraindicated in patients with severe aortic stenosis.

### 4.3. Limitations

This study included a moderate-sized cohort, and we assumed all continuous variables to be non-parametric and presented them as median and interquartile rather than mean and standard deviation. Not all patients completed the 4-year observation, which reduces the statistical power of this study. We attempted to adjust for potential confounders to examine the prognostic impact of psoas muscle mass index, but we may have missed other uninvestigated potential confounders that also have a considerable prognostic impact, including aortic valve calcification and renal function [27]. We measured psoas muscle mass only once before TAVR, and the prognostic impact of its trend was not evaluated. The impact of aggressive cardiac rehabilitation on increasing psoas muscle mass and improving sarcopenia remains uncertain.

## 5. Conclusions

Lower psoas muscle mass index, a recently introduced objective marker of sarcopenia, was associated with mid-term mortality after TAVR in the elderly cohort. The clinical application of psoas muscle mass index-guided management in TAVR candidates requires further evaluation.

## Figures and Tables

**Table 1:** Baseline characteristics at index discharge.

	Total (N = 322)	Deceased (N = 36)	Alive (N = 286)	*p* Value
Demographics				
Age, years	85 (83, 88)	86 (84, 89)	85 (82, 88)	0.12
Male sex	95 (30%)	13 (36%)	82 (29%)	0.23
Body surface area, m^2^	1.39 (1.29, 1.52)	1.41 (1.37, 1.51)	1.30 (1.25, 1.49)	0.30
Body mass index	21.7 (19.4, 24.4)	21.8 (19.7, 23.7)	21.7 (19.2, 24.4)	0.85
Systolic BP, mmHg	117 (106, 128)	109 (106, 118)	112 (105, 119)	0.49
Pulse rate, bpm	70 (63, 78)	72 (63, 82)	71 (62, 79)	0.40
STS score	4.6 (3.9, 6.1)	6.6 (3.8, 9.7)	4.3 (3.2, 5.8)	<0.001 *
Comorbidity				
Coronary artery disease	87 (27%)	6 (17%)	81 (28%)	0.096
Peripheral artery disease	71 (22%)	15 (42%)	56 (20%)	0.004 *
Diabetes mellitus	58 (18%)	3 (8%)	55 (19%)	0.078
Atrial fibrillation	42 (13%)	9 (25%)	33 (12%)	0.029 *
Laboratory data				
Hemoglobin, g/dL	10.4 (9.6, 11.2)	10.5 (8.4, 11.3)	10.6 (10.0, 11.0)	0.46
Serum albumin, g/dL	3.4 (3.1, 3.6)	3.5 (3.0, 3.6)	3.5 (3.2, 3.9)	0.093
Serum sodium, mEq/L	139 (137, 141)	140 (137, 142)	139 (138, 140)	0.95
Total cholesterol, mg/dL	154 (137, 172)	144 (97, 176)	163 (145, 175)	0.33
eGFR, mL/min/1.73 m^2^	51 (37, 64)	37 (33, 46)	57 (37, 67)	0.055
Plasma BNP, pg/mL	101 (55, 210)	156 (73, 178)	103 (41, 157)	0.012 *
Echocardiography				
LVDd, mm	45 (40, 50)	47 (43, 53)	42 (39, 50)	0.96
LVEF, %	64 (56, 70)	68 (45, 71)	63 (58, 70)	0.77
Left atrial diameter, mm	41 (36, 52)	41 (36, 45)	38 (35, 47)	0.98
AV peak velocity, m/s	2.2 (1.8, 2.4)	2.2 (1.9, 2.5)	2.1 (1.7, 2.4)	0.65
AV mean velocity, m/s	11 (7, 12)	10 (6, 13)	11 (8, 12)	0.77
AV area, cm^2^	1.4 (1.2, 1.6)	1.7 (1.3, 2.0)	1.4 (1.2, 1.6)	0.52
Frailty data				
MMS	26 (23, 28)	22 (19, 279	27 (24, 28)	0.076
CSHA scale	4 (3, 4)	5 (4, 6)	3 (3, 4)	0.002 *
SAS, Mets	5.0 (4.0, 5.8)	4.5 (4.5, 5.3)	5.5 (4.0, 6.0)	0.89
Barthel index	100 (95, 100)	95 (73, 100)	100 (100, 100)	0.11

BP, blood pressure; eGFR, estimated glomerular filtration ratio; BNP, B-type natriuretic peptide; LVDd, left ventricular end-diastolic diameter; LVEF, left ventricular ejection fraction; AV, aortic valve; MMS, mini-mental state; CSHA, Canadian Study of Health and Aging; SAS, specific activity scale. Continuous variables are stated as median and interquartile and compared between the two groups using Mann–Whitney U test. Categorical variables are stated as numbers and percentage and compared between the two groups using Fischer’s exact test. * *p* < 0.05.

**Table 2:** Impact of baseline characteristics on 4-year mortality.

	Univariable Analysis		Multivariable Analysis	
	Hazard Ratio (95%CI)	*p* Value	Hazard Ratio (95%CI)	*p* Value
STS score	0.99 (0.98–1.01)	0.87	NA	NA
Peripheral artery disease	2.05 (1.05–3.98)	0.035 *	1.55 (0.75–3.21)	0.24
Atrial fibrillation	2.47 (1.16–5.26)	0.019 *	2.04 (0.93–4.45)	0.075
Logarithm of BNP, pg/mL	2.54 (1.20–5.39)	0.015 *	1.92 (0.84–4.41)	0.12
CSHA scale	1.58 (0.97–2.12)	0.076	NA	NA
Psoas muscle mass index, ×10 cm^3^/m^2^	0.89 (0.79–0.99)	0.041 *	0.88 (0.79–0.99)	0.044 *

CI, confidence interval; BNP, B-type natriuretic peptide; CSHA, Canadian Study of Health and Aging. Variables significant in the univariable analyses were included in the multivariable analysis. * *p* < 0.05.

**Table 3:** Baseline characteristics that were potentially associated with sarcopenia.

	Small Psoas Muscle Mass Index	Large Psoas Muscle Mass Index	*p* Value
Age, years	85 (82, 89)	86 (82, 89)	0.076
Body mass index	22.6 (20.0, 24.5)	20.9 (19.0, 23.5)	0.004 *
Hemoglobin, g/dL	10.4 (9.6, 11.5)	10.6 (10.0, 10.9)	0.006 *
Serum albumin, g/dL	3.5 (3.2, 3.7)	3.5 (3.4, 4.0)	0.71
Total cholesterol, mg/dL	154 (131, 164)	166 (146, 178)	0.007 *
MMS	24 (20, 27)	28 (27, 30)	0.008 *
CSHA score	4 (3, 5)	4 (3, 4)	0.10
SAS, Mets	4.5 (3.9, 5.5)	5.5 (4.3, 6.0)	0.053
Barthel index	100 (95, 100)	100 (95, 100)	0.73
Psoas mass index, ×10 cm^3^/m^2^	9.4 (8.1, 10.3)	14.1 (12.1, 17.7)	<0.001 *

MMS, mini-mental state; CSHA, Canadian Study of Health and Aging. Small psoas muscle mass was defined as small psoas muscle mass index <10.7 × 10 cm^3^/m^2^. Continuous variables are stated as median and interquartile and compared between the two groups using Mann–Whitney U test. Categorical variables are stated as numbers and percentage and compared between the two groups using Fischer’s exact test. * *p* < 0.05.

## Data Availability

Data are available upon reasonable request from the corresponding author.
